# Case of Cornual Uterine Rupture in Subsequent Pregnancy Following Laparoscopic Removal of Cornual Ectopic Pregnancy

**DOI:** 10.7759/cureus.49766

**Published:** 2023-12-01

**Authors:** Sofia Malik, Asma Fahad

**Affiliations:** 1 Obstetrics and Gynecology, Dubai Academic Health Corporation, Dubai, ARE

**Keywords:** hemoperitoneum in pregnancy, placental abruption, high risk pregnancy, cornual ectopic pregnancy, uterine rupture

## Abstract

Uterine rupture during pregnancy is a critical obstetric complication associated with maternal and fetal mortality and morbidity. We present a case of uterine rupture at 27 weeks of gestation, following a previous cornual pregnancy managed laparoscopically. Our case report underlines the importance of multilayered uterine wall repair following the resection of cornual ectopic pregnancy to avoid such potentially catastrophic sequelae.

## Introduction

Cornual gestation is one of the most hazardous types of ectopic gestation and accounts for 2-4% of ectopic pregnancies a mortality rate in the range of 2.0-2.5% [1]. Some surgeons have suggested reconstruction of the uterine wall defect at the time of surgical management of the cornual ectopic pregnancy to reinforce the defective area in the uterine wall.

Uterine rupture during pregnancy is a critical obstetric complication associated with maternal and fetal mortality and morbidity. It can occur at the site of a previous interstitial pregnancy [2].

Here we present a case of uterine rupture at 27 weeks of gestation, following a previous cornual pregnancy (interstitial portion of the fallopian tube) managed laparoscopically. Our case report underlines the importance of multilayered uterine wall repair following the resection of cornual ectopic pregnancy to avoid such potentially catastrophic sequelae.

This case has been presented as a poster presentation at the RCOG World Congress 2023.

## Case presentation

We present the case of a 37-year-old female, para 4, with all previous vaginal deliveries at term.

**Figure 1:**
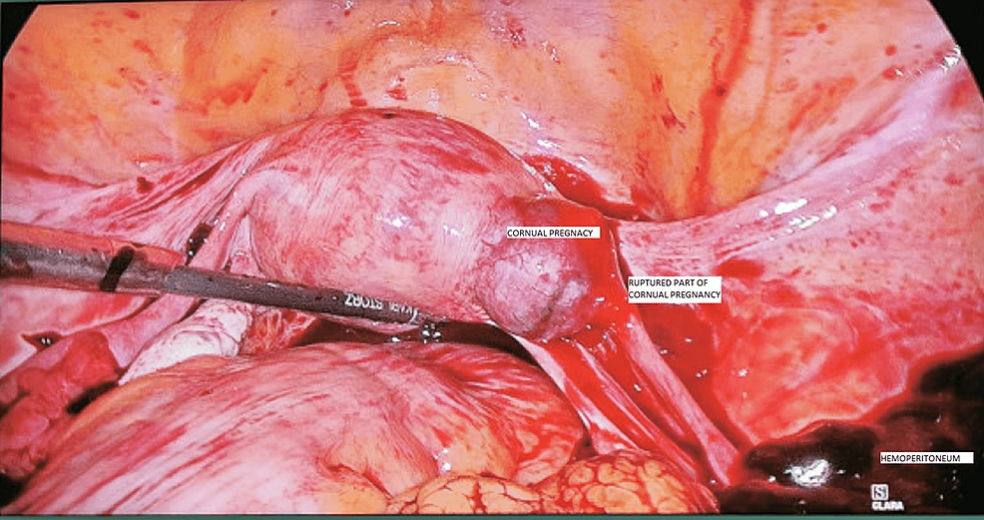
Right cornual pregnancy with hemoperitoneum

She presented to Latifa Hospital at 5 weeks of gestation (by last menstrual period, LMP), was diagnosed with a ruptured ectopic pregnancy, and was taken for laparoscopic management. Intraoperatively she was found to have a 3-liter hemoperitoneum with a right cornual pregnancy (interstitial part of the fallopian tube) with active bleeding (Figure 1). The corneal pregnancy was excised along with the right tube, and the site of the cornual pregnancy was sutured interruptedly with 2-0 Vicryl.

Histopathology report showed the body of the fallopian tube with cornual dilatation measuring 8.0 cm in length.

The patient conceived after 8 months, despite being counseled for contraception. She was followed by obstetricians in our hospital's antenatal clinic since early pregnancy.

She attended the obstetric emergency department at 27 weeks of gestation with severe abdominal pain. On examination, she was afebrile and tachycardic with normal blood pressure and respiratory rate. Her pain score was 9. Abdominal examination showed tenderness over the right fundal area and fullness of the right hypochondrium. The fetal assessment showed fetal bradycardia at 60 beats per minute. A provisional diagnosis was made of uterine rupture with a differential diagnosis of placental abruption, and the patient was taken for an emergency cesarean section after giving consent.

**Figure 2:**
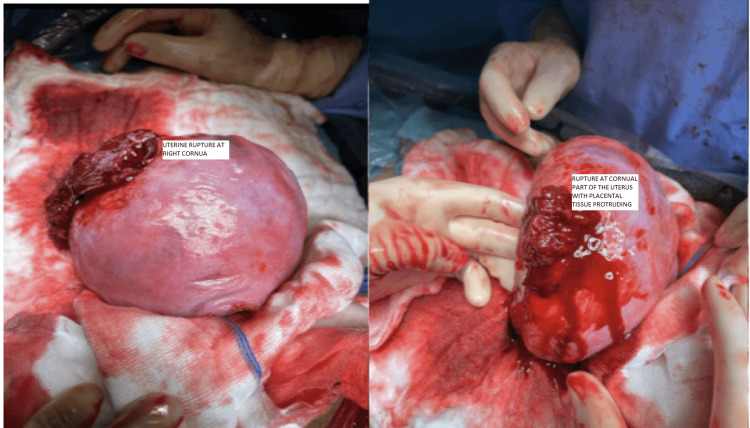
Uterine rupture at the right cornua with placental tissue

Intraoperatively she was found to have frank hemoperitoneum with uterine rupture around 4-5 centimeters in the right cornual area with protruding placental tissue (Figure 2).

The fetus weighed 1300 grams and was delivered and given to the neonatologist.

The fetus was born with APGAR scores of 3 and 7 at 1 and 5 minutes respectively. He was in the NICU for 58 days under the neonatology team and was discharged in good and stable condition. 

The on-call consultant was called for and the uterine rupture site was sutured in three layers by interrupted sutures with 2-0 Vicryl. Hemostasis was maintained. The uterus was closed in three layers. Hemostasis was secured. Blood clots of around 1.5 liters were removed from the peritoneal cavity. Closure was performed in a routine manner. The patient was transfused with one unit of packed red blood cells intraoperatively. Estimated blood loss was calculated as 2 liters.

Postoperatively, the patient and her partner were counseled regarding diagnosis, treatment, management, and contraception and on adequate time intervals between pregnancies. The patient recovered well and was discharged in stable condition.

## Discussion

A concern regarding future pregnancy after surgical management of cornual pregnancy is the rupture of the interstitial portion of the fallopian tube (uterine rupture) [1], which may be due to a defect in the uterine wall [3]. Uterine rupture occurs at a frequency of <1% in women with a previously scarred uterus, with retrospective studies quoting rates of approximately 0.65% [4].

There are several case reports of uterine rupture in the third trimester following cornual resection due to ectopic pregnancy. Košec et al. (2021) have published a case of uterine rupture at 32 weeks following a laparotomy repair to interstitial pregnancy, which had been preceded by a laparoscopic salpingectomy 5 years prior [5]. A similar case was presented by Abbas et al. (2016) concerning cornual rupture at 39 weeks of gestation with a history of laparoscopic removal of ectopic pregnancy [6].

The usual presentation in uterine rupture is restlessness and constant pain in the lower uterus. Clinically, there may be tachycardia and cardiotocography (CTG) abnormalities, such as sudden and persistent bradycardia consistent with fetal compromise. Diagnosis of uterine rupture warrants resuscitation and exploratory laparotomy. The importance of immediate senior involvement and teamwork cannot be overemphasized [7].

In our case, the prompt diagnosis and management of uterine rupture prevented maternal and fetal mortality.

## Conclusions

Pregnancy following the removal of interstitial ectopic pregnancy should be classified as high risk, and the presentation of abdominal pain in such cases should be promptly assessed and managed, keeping in mind the risk of uterine rupture. This case study underscores the importance of proper repair of defects in the uterine wall at the time of resection/removal of cornual/interstitial ectopic pregnancy.
